# Are Empty-Nest Elders Unhappy? Re-examining Chinese Empty-Nest Elders’ Subjective Well-Being Considering Social Changes

**DOI:** 10.3389/fpsyg.2020.00885

**Published:** 2020-05-20

**Authors:** Yan Zhang

**Affiliations:** School of Psychology, Central China Normal University, Wuhan, China

**Keywords:** empty-nest, living arrangement, rural-urban gap, social individualization, China

## Abstract

Aging, the one-child policy, and migration have altered Chinese family structure and the number of empty-nest elders is increasing. Since living without children runs in the opposite direction of filial piety, empty-nest elders have typically been negatively viewed and depicted as unhappy. However, individualization and the unbalanced development of China may decrease the impact of children but increase the impact of the spouse and rural-urban gaps on elders’ well-being. Therefore this study re-examined the subjective well-being of empty-nest elders considering these social changes. Participants (*N* = 765; age range = 60–94 years, *M*_*age*_ = 70.25 years, *SD*_*age*_ = 7.85; men = 45%) were recruited from two large cities, two small cities, and two rural areas in China. Elders’ subjective well-being was measured by the Memorial University of Newfoundland Scale of Happiness–Chinese version. Results showed that participants were happy on average; empty-nest elders were not unhappier than non-empty-nest elders. Elders living without a spouse and rural elders had a high risk of being unhappy. Policymakers should thus shift their attention from empty-nest families to the widowed and rural elders.

## Introduction

China is experiencing huge social changes. First, the Chinese society is aging. From 2000 to 2018 the number of senior citizens in China (aged ≥ 60 years) increased from 129 million to 249 million (i.e., from 10.5 to 17.9% of the total population; National Bureau of Statistics, 2010; Ao et al., 2015). Further, although many elders require care, the one-child policy enforced in China for 30 years resulted in many young Chinese adults not having siblings to share the elderly care obligations. Additionally China is moving toward urbanization. In 2014, 168 million workers had transitioned from rural to urban areas (Ao et al., 2015). China is also ranked the fourth largest migrant-sending country worldwide (United Nations, 2013).

The consequences of aging, the one-child policy, and migration are the erosion of the traditional family structure and the expansion of the population of empty-nest elders (Xu et al., 2014; Fang et al., 2015). It was estimated that, by the end of 2020, Chinese empty-nest elders will reach 118 million, accounting for about 50% of the total elderly population (The State Council of China, 2017). However, Chinese empty-nest families have been far less studied than Western families (Shek, 2006).

The word “empty-nest” comes from family life cycle theory (Duvall, 1957). In this theory, the stage from the time the last child departs from home to one’s partners’ death is referred to as the “empty-nest stage.” However, the label “empty-nest elders” used by Chinese scholars, refers particularly to *elders* living without children, *regardless of whether one’s spouse is alive*. The reason for the different usage of the word “empty-nest” may be that it was introduced in China in the 1990s (Wang, 1995). At that time, Chinese citizens regarded the father–son relationship as the most important familial relationship and valued filial piety (Hsu, 1948; Shek, 2006).

Filial piety (*xiao*) requires children to ensure the continuance of their parents’ happiness – not only by respecting older generations but also by taking care of old parents (Zeng and George, 2010). Although in many Western countries it is normal for elders to live without children nearby, in China this was not the case. Elders used to live with several generations under one roof and rely on children, especially sons, for support (Sung, 2001; Zeng and George, 2010).

Living in an empty-nest in one’s late life runs in the opposite direction of traditional culture; consequently, empty-nest elders were negatively viewed by the public and were commonly depicted as unhappy, lonely, and depressed in the mass media (e.g., eChinacities, 2014; CHINADAILY, 2015). Some researchers (e.g., Borland, 1982; Mitchell and Lovegreen, 2009) claimed that parents, especially mothers, experienced many negative emotions, such as depression, when their children all left home. They named this phenomenon “empty-nest syndrome.” Nonetheless Bouchard (2014) reviewed research about empty-nesters and subjective well-being (SWB) and revealed inconsistent results. Some empty-nest women experienced a sense of loneliness and loss, however, few parents displayed empty-nest syndrome. Further, for most parents, their SWB increased or did not change after their children left home.

Research concerning Chinese empty-nest elders’ SWB has also failed to yield consistent conclusions. Some researchers found that, compared to non-empty-nest elders (elders living with children), empty-nest elders had lower SWB (Silverstein et al., 2006), worse mental health (Liu et al., 2007), and had higher loneliness (Cheng et al., 2015) and depression (Zhai et al., 2015). In contrast, Lin et al. (2009) found that empty-nesters did not differ from non-empty-nesters in loneliness, and some other researchers found that empty-nest elders displayed higher SWB (Liu et al., 2014) and life satisfaction (Sun, 2010; Poulin et al., 2014) than did non-empty-nest elders.

Why do the results vary and why do some findings contradict the negative descriptions of empty-nest elders? There may be two reasons. First, Chinese society is individualizing: the importance of the individual is increasing; the pursuit of privacy, independence, choice, and personal happiness is popularizing; and the importance of filial piety is decreasing (Beck and Beck-Gernsheim, 2001; Ng et al., 2002; Yan, 2011). Thus, the individualization of Chinese society may decrease the impact of children on elders’ SWB. Indeed, Miller (2007) found that an increasing number of elders are unwilling to live with children and can live independently and happily. In this context, *when* studies were conducted becomes important. In more recent studies, living in an empty-nest may have a smaller effect on elders’ SWB as compared to studies from several years ago.

Another characteristic of society individualization is that the marital relationship is replacing the father–son relationship as the most important relationship in families (Yan, 2011). In an individualizing society, living with a spouse may have a larger effect on elders’ SWB compared to living with children. Ren and Treiman (2015) found that, compared to those living with their spouse, elders living with grown children were less happy, had less life satisfaction, and were more depressed. However, the Chinese label “empty-nest elders” ignored the impact of a spouse. Wang et al. (2014) found that, for the widowed, co-residence with adult children was associated with better SWB than living alone, however, for married elders, co-residence did not bring about additional benefits concerning SWB. Therefore, mixing married and unmarried elders into one category may bias the results, and empty-nest elders and non-empty-nest elders may be incomparable if marital status differs or remains statistically uncontrolled.

Second, the vulnerability of empty-nest elders, especially rural empty-nest elders, may not be caused by living arrangement but by the unbalanced development of different regions. At the 19th CPC National Congress, President Xi said that the principal contradiction facing Chinese society in the new era is that between unbalanced and inadequate development and people’s ever-growing need for a better life (XINHUA, 2017). Geographically, China is large, and different regional policies and the increasing urban–rural gap may foster regional and urban–rural disparities in elders’ SWB. Some researchers found that, compared to rural elders, urban elders had higher life satisfaction (Wang et al., 2011; Li et al., 2014), better mental health (Tian et al., 2015), and better quality of life (Zhou et al., 2010). Therefore, researchers who collected data from more than one region may have yielded varied results because of the interaction between regional differences and living arrangements (e.g., Ma et al., 2012).

Consequently, this study re-examined Chinese empty-nest elders’ SWB by considering social changes, and addressed whether individualization and the unbalanced development of China has decreased the impact of children but increased the impact of the spouse and rural-urban gaps on elders’ well-being.

## Materials and Methods

### Design and Hypotheses

This study hypothesized that *empty-nest elders may be as happy as non-empty-nest elders (Hypothesis 1)*, therefore, a control group was utilized: non-empty-nest elders (i.e., elders living without children). It was also hypothesized that *having a spouse would have a positive effect on SWB (Hypothesis 2)*. Since simply grouping participants as empty-nest or non-empty-nest elders did not account for marital status, empty-nest elders and non-empty-nest elders were further divided into five categories: elders living alone, elders living with a spouse only, elders living with children only, elders living with a spouse and children, and elders living with others. The third hypothesis *(Hypothesis 3)* was that *rural elders would have a lower SWB than would urban elders*; thus, the SWB of elders from large cities, small counties, and rural areas were also compared.

### Recruitment of Participants

This study used multistage sampling strategies. In the first stage, to explore regional differences, participants were recruited from three regional levels: large cities, small counties, and rural areas. Then, based on the accessibility of participants and understandability of dialects, a large city from northern and southern China (i.e., Beijing and Shanghai, respectively) and a small county from northern and southern China (Linqu of Shandong Province and Liling of Hunan Province, respectively) were selected. Rural areas were villages under the jurisdiction of the two small counties.

The two large cities – Beijing and Shanghai – have a similar population (in 2018, 21.54 million and 24.24 million, respectively) and economic development level (gross domestic product (GDP) in 2018 was 3.03 trillion yuan and 3.27 trillion yuan, respectively). The two small counties Linqu and Liling are of similar population (total population of 2018 was 0.93 million and 1.05 million, respectively) and economic development level (GDP in 2018 was 305.51 billion yuan and 580.8 billion yuan, respectively).

In the second stage a cluster stratified sampling strategy was used. Eight communities were randomly selected from each large city, and eight communities and eight villages were randomly selected from each small county.

In the third stage participants were recruited from selected communities/villages by an accidental sampling strategy. Nine-hundred elders aged 60 years or older (150 from each city/county/rural area) were targeted for enrollment. The number of participants to be recruited from each community/village was decided by their proportion of the total population. For example, if a specific community in Beijing had a population of 2,000, and all of the selected communities in Beijing had a total population of 20,000 people, since 150 participants from Beijing were targeted, 15 participants should be recruited from the specific community. Participants with cognitive, emotional, hearing, or speaking difficulties that hindered them finishing questionnaires were excluded. In total 765 participants completed the questionnaires (effective response rate = 85%).

### Participants’ Characteristics

Participants’ ages ranged from 60 to 94 years (*M* = 70.25 years, *SD* = 7.85). 345 were men (45%). Among all participants, 300 (39.2%) were from large cities, 189 (24.7%) were from small counties, and 276 (36.1%) were from rural areas. There were no sex or age differences among elders between different regions.

Participants from large cities had the highest education level: over 80% had an education level of junior high school or above, while over half from small counties or rural areas had an education level of elementary or below. Participants from large cities also had the best financial status compared to their counterparts; specifically, 90% had a retirement pension, while this ratio for participants from small counties and from rural areas was 54.5 and 8%, respectively. A retirement pension is a major income source for elders, and over half the participants from large cities earned more than 5,000 yuan (about 700 U.S. dollars) per month, while most participants from small counties had an income around 2,000 to 3,000 yuan (about 280 to 430 U.S. dollars) per month. Over half the rural participants earned less than 500 yuan (about 70 U.S. dollars) per month. Correspondingly, about 50% of the urban participants perceived their income to be adequate, while over 50% of the rural participants perceived their income to be insufficient.

The health status of elders from small counties was the best: they had fewer illnesses, including chronic illnesses, compared to the other elders. More participants from large cities had critical illnesses than did participants from other areas. Moreover, more participants from large cities were empty-nest elders (61.0%), while this ratio for participants from small counties and rural areas was 41.8 and 35.5%, respectively.

For participants from large cities, 20% of them lived with a spouse only, while more participants from small counties and rural areas tended to live with a spouse and children (11.1 and 15.4%, respectively). In addition, more rural elders were currently unmarried, and a higher ratio were living with children (7.2%) as compared to urban elders (around 3% for both elders from large cities or small counties). Compared with urban elders, more rural elders had a religion and were farmers previously. Rural elders also had more children than did urban elders. Notably, all participants had medical insurance (Table 1).

**TABLE 1:** Sample characteristics by living regions.

	Coding strategy	Total(*N* = 765)	Living regions			
			Rural areas (*N* = 276)	Small counties (*N* = 189)	Large cities (*N* = 300)	χ^2^/*F*
**Sex**						
Men, %	women = 0, Men = 1	45.0	43.5	46.0	45.0	0.31
**Age, M (SD)**		70.25 (7.85)	70.21 (7.88)	70.22 (7.29)	70.30 (0.19)	0.01
**Religion**						
Yes, %	No = 0, yes = 1	17.1	27.9	10.1	11.7	35.53***
**Education**						186.55***
Elementary and below, %	= 1	43.1	65.6	57.1	13.4	
Junior high, %	= 2	34.0	21.4	27.5	49.8	
High, %	= 3	16.1	11.2	11.1	23.7	
College and above, %	= 4	6.8	1.8	4.2	13.0	
**Previous vocation**						429.56***
No, %	= 0	2.0	1.8	4.2	0.7	
Farmer, %	= 1	42.4	84.8	45.0	1.7	
Worker, %	= 2	36.8	9.1	29.6	66.9	
Others, %	= 3	18.8	4.3	21.2	30.8	
**Marital status**						
Married, %	unmarried = 0, married = 1	79.4	73.4	83.1	82.6	10.27**
**Number of children, M (SD)**	2.59 (1.38)	3.23 (1.29)	2.91 (1.28)	1.79 (1.03)	113.94***	
**Health status**						
**Number of illness, M (SD)**	1.56 (1.33)	1.50 (1.18)	1.05 (1.04)	1.94 (1.49)	28.30***	
Have chronic illness, %	No = 0, Yes = 1	72.3	76.1	60.8	76.0	16.40***
Have critical illness, %	No = 0, Yes = 1	36.2	32.2	32.8	42.0	7.18*
Have medical Insurance, %	No = 0, Yes = 1	100.0	100.0	100.0	100.0	/
**Financial status**						
Income per month						644.76***
≤ = 100, %	= 1	9.8	22.9	6.3	0.0	
101∼500, %	= 2	17.0	35.6	15.9	0.7	
501∼1000, %	= 3	10.3	18.5	13.2	1.0	
1001∼2000, %	= 4	11.1	13.1	22.2	2.3	
2001∼3000, %	= 5	13.6	6.9	23.8	13.3	
3001∼5000, %	= 6	14.0	2.5	10.6	26.7	
5001∼10,000, %	= 7	22.1	0.4	7.9	51.0	
≥ = 10001, %	= 8	2.0	0.0	0.0	5.0	
Retirement pension						
Have, %	No = 0, have = 1	51.6	8.0	54.5	90.0	388.14***
**Income adequacy**					94.56***	
No, %	= 1	33.3	51.4	22.2	23.7	
Moderate, %	= 2	34.1	35.5	31.2	34.7	
Adequate, %	= 3	32.5	13.0	46.6	41.7	
**Empty-nest**						
Yes, %	No = 0, Yes = 1	47.1	35.5	41.8	61.0	40.29***
**Co-residence**						49.42***
Alone, %	= 1	7.3	2.4	1.4	3.5	
Spouse, %	= 2	39.7	10.5	8.9	20.4	
Children, %	= 3	12.8	7.2	2.7	2.9	
Spouse and children, %	= 4	38.2	15.4	11.1	11.6	
Others, %	= 5	2.0	0.7	0.5	0.8	

*p < 0.05; **p < 0.01; ***p < 0.001.

Nearly half the participants (360, 47.1%) were empty-nest elders. Empty-nest elders and non-empty-nest elders did not significantly differ in sex, age, religion, or health status. However, empty-nest elders had a higher education level, more children, and more income per month than did non-empty-nest elders. Further, compared to non-empty-nest elders, more empty-nest elders were previously workers, currently married, had a retirement pension, and perceived their income to be adequate (Table 2).

**TABLE 2:** Sample characteristics by living arrangements.

	Empty-nest			Co-residence					
	
	Non-empty-nest elders (*N* = 405)	Empty-nest elders (*N* = 360)	χ^2^/*t*	Living alone (*N* = 56)	With spouse only (*N* = 304)	With children only (*N* = 98)	With spouse and child (*N* = 292)	With others (*N* = 15)	χ^2^/*F*
**Sex:** men, %	44.90	44.40	0.02	39.3	45.4	36.7	46.2	73.3	8.49
**Age, M (SD)**	70.75 (8.23)	69.69 (7.38)	1.87	74.29 (8.22)	68.84 (6.91)	75.96 (9.06)	69.02 (7.15)	70.27 (8.29)	23.322***
**Religion:** Yes, %	18.80	15.30	1.63	19.6	14.5	17.3	19.5	13.3	3.11
**Education**			35.67***						80.49***
Elementary and below, %	53.10	31.80		50.0	28.4	76.5	44.9	60.0	
Junior high, %	28.60	40.10		35.7	40.9	17.3	32.9	20.0	
High, %	12.60	20.10		8.9	22.1	5.1	15.4	6.7	
College and above, %	5.70	8.10		5.4	8.6	1.0	6.8	13.3	
**Previous vocation**			35.86***						56.56***
No, %	1.70	2.20		3.6	2.0	3.1	1.0	6.7	
Farmer, %	52.50	31.10		28.6	31.6	64.3	49.1	40.0	
Worker, %	29.70	44.70		53.6	43.1	24.5	30.6	46.7	
Others, %	16.10	21.90		14.3	23.4	8.2	19.2	6.7	
**Marital status:** Married, %	72.80	86.10	20.30***	11.3	100.0	0.0	100.0	20.0	714.89***
**Number of children, M (SD)**	2.77 (1.37)	2.38 (1.31)	3.91***	2.73 (1.47)	2.32 (1.27)	3.53 (1.48)	2.57 (1.23)	1.73 (1.39)	17.54***
**Health status**									
Number of illness, M (SD)	1.48 (1.28)	1.66 (1.37)	−1.82	1.88 (1.25)	1.62 (1.39)	1.50 (1.18)	1.49 (1.33)	1.13 (0.92)	1.55
Have chronic illness, %	71.10	73.60	0.60	83.9	71.7	75.5	69.5	73.3	5.55
Have critical illness, %	34.60	38.10	1.00	42.9	37.2	33.7	35.3	26.7	2.17
**Financial status**									
Income per month			68.97***						176.47***
≤100, %	14.90	4.20		8.9	3.3	41.8	6.2	6.7	
101∼500, %	21.30	12.20		14.3	11.8	18.4	22.3	20.0	
501∼1000, %	12.40	8.10		8.9	7.9	7.1	14.1	13.3	
1001∼2000, %	11.40	10.80		12.5	10.5	9.2	12.0	13.3	
2001∼3000, %	13.60	13.60		28.6	10.9	13.3	13.7	13.3	
3001∼5000, %	11.60	16.70		19.6	16.1	7.1	12.4	26.7	
5001∼10000, %	13.40	31.90		7.1	36.5	3.1	17.2	6.7	
≥ 10001, %	1.50	2.50		0.0	3.0	0.0	2.1	0.0	
Retirement pension									
Have, %	40.70	63.90	40.89***	55.4	65.5	24.5	46.2	40.0	56.72***
Income adequacy			14.63***						18.08*
No, %	38.80	27.20		28.6	27.0	40.8	38.0	40.0	
Moderate, %	33.80	34.40		42.9	32.9	33.7	33.9	33.3	
Adequate, %	27.40	38.30		28.6	40.1	25.5	28.1	26.7	

*p < 0.05; **p < 0.01; ***p < 0.001.

For further classification, 56 (7.3%) of the elders were living alone, 304 (39.7%) were living with a spouse only, 98 (12.8%) were living with children only, 292 (38.2%) were living with a spouse and children, and 15 (2.0%) were living with other people. Among them, sex, religion, and health status did not significantly differ. Participants living without a spouse (including living alone and living with children only) were older and had a lower education level than did participants living with a spouse (including living with a spouse only and living with children and a spouse). More participants living without children (including living alone and living with a spouse only) were workers previously and had better financial status than did participants living with children (including living with children only and living with children and a spouse, Table 2).

### Measures

#### Dependent Variables

SWB was measured by the Memorial University of Newfoundland Scale of Happiness (MUNSH), developed by Kozma and Stones (1983), which has good psychometric properties when used with elderly individuals (Andrews and Robinson, 1991). The translated Chinese version of the MUNSH has been commonly used in Chinese studies and has high reliability and validity (e.g., Deng et al., 2010). In this study, the MUNSH scale also had a high reliability (α = 0.89).

The MUNSH is a 24-item self-report scale that includes five items measuring positive attitudes (PA), five items measuring negative attitudes (NA), seven items measuring positive experiences (PE), and seven items measuring negative experiences (NE). There are three response options: “yes” is scored as 1, “uncertain” is scored as 0, and “no” is scored as -1. When computing the subscale scores and total scale score, scores are converted to a positive range, therefore, the PA and NA subscales range from 0 to 10 and the PE and NE subscales range from 0 to 14. The range for the total scale score is 0–48. A total score of 32 or above is considered a high level of happiness. The higher the total score, the higher participants’ SWB.

#### Independent Variables

The variable “living regions” had three levels: large city, small county, and rural area, representing the regions each participant was recruited from. Living arrangements were divided in two ways. First, it was divided into two categories: empty-nest and non-empty-nest. Second, it was divided into five categories: living alone, living with a spouse only, living with children only, living with a spouse and children, and living with others. To distinguish, the first method of classification was termed the “empty-nest” variable, and the second method of classification was termed the “co-residence” variable.

#### Control Variables

Control variables included sociodemographic variables that were found to be highly related to SWB (Diener, 1999), including the SWB of elders (Pinquart and Sorensen, 2000): sex, age, religion (religious or not), education (elementary and below, junior high, high school, and college and above), previous vocation (no vocation, farmer, worker, and others), current marital status (unmarried, which includes unmarried, widowed and divorced; and married, which includes married and remarried), number of children, health status, and financial status.

Health status is difficult to evaluate with only one index, therefore, four variables were used to estimate participants’ overall health status: number of illnesses (participants were asked to name/write down all the diseases they have now and the total number was counted), if they have a chronic illness, if they have a critical illness, and if they have medical insurance.

Moreover, financial status is difficult to evaluate by only one index, therefore, three variables were used to estimate participants “overall financial status”: income per month (divided into eight levels from ≤ 100 yuan per month to ≥ 10,001 yuan per month), if they have a retirement pension (this variable was chosen because a retirement pension is a main economic resource for elders), and if they perceived their income to be adequate (this variable was chosen to test participants’ subjective financial status).

Among all the control variables, age, number of illnesses, and number of children were continuous variables; income was treated as a continuous variable. Other control variables were binary or categorical variables. Categorical variables were coded into dummy variables in analyses. Specific coding strategies are shown in Table 1.

### Statistical Analyses

SPSS 22.0 (IBM, Armonk, NY, United States) was used for data management and analyses. First, the effect of living regions and living arrangements on SWB were analyzed with an analysis of variance. Then, a general linear model was used to analyze the interaction effect of living regions and living arrangements on SWB to test whether the different SWB between empty-nest elders and non-empty-nest elders was affected by the disproportion rate of empty-nest elders/non-empty-nest elders among living regions. Finally, ordinary least squares regression analyses were used to predict elders’ SWB with a set of hierarchical equations.

In regression Model 0, control variables were entered, except current marital status and the three variables representing financial status (income per month, retirement pension, and income adequacy). The purpose was to determine spouses’ role (reflected by current marital status) and the role of living region (related to financial status) on elders’ SWB, therefore they were controlled in later models.

Based on Model 0, empty-nesters was entered into Model 1. Then, current marital status was entered in Model 2 and financial status in Model 3. Finally, living region was entered in Model 4.

To further explore the role of spouses, SWB was estimated again by using the independent variable “co-residence.” The procedure was the same as before: co-residence was entered in Model 5 based on Model 0. Current marital status was not controlled because it was highly related to co-residence. Financial status was then entered in Model 6. Finally, living regions was entered in Model 7.

### Ethical Considerations

The need for ethical approval was waived by the university. No personal identifiable information was gathered, such as name, living address, ID, and telephone number. Questionnaires were completed individually in face-to-face interviews, which lasted from 10 to 30 min. After finishing the questionnaire, each participant was given a small gift (worth about one dollar).

## Results

### Overall SWB

The SWB score of all participants ranged from 1 to 48 (*M* = 33.84, *SD* = 10.07), suggesting that Chinese elders were happy on average (i.e., >32). The PA scores ranged from 5 to 15, *M* = 12.22, *SD* = 2.58; the NA scores ranged from 5 to 15, *M* = 8.13, *SD* = 2.80; the PE scores ranged from 7 to 21, *M* = 16.74, *SD* = 3.06; and the NE scores ranged from 7 to 21, *M* = 10.99, *SD* = 3.49, indicating that Chinese elders had more positive (vs. negative) attitudes and experiences.

### Living Regions and SWB

Participants from different living regions had different SWB [*F*(2, 762) = 36.724, *p* < 0.001]. Participants from large cities had the highest SWB, *M* = 36.91, *SD* = 8.58; while participants from rural area had the lowest SWB, *M* = 30.08, *SD* = 10.78. Rural participants had significantly lower SWB than did participants from small counties and large cities. Participants from small counties had significantly lower SWB than did participants from large cities (Table 3).

**TABLE 3:** Effect of living regions and living arrangements on subjective well being.

	N	SWB *M* (*SD*)	PA *M* (*SD*)	NA *M* (*SD*)	PE *M* (*SD*)	NE *M* (*SD*)
**Living regions**						
Rural areas	276	30.08 (10.78)	11.45 (2.88)	9.24 (2.88)	15.89 (3.10)	12.03 (3.65)
Small counties	189	34.47 (9.41)	12.55 (2.62)	7.81 (2.69)	16.69 (2.90)	10.96 (3.23)
Large cities	300	36.91 (8.58)	12.71 (2.05)	7.30 (2.46)	17.55 (2.90)	10.05 (3.24)
F-value		36.72***	20.14***	39.52***	22.21***	24.69***
**Empty-nest**						
No	405	32.71 (10.57)	32.71 (10.57)	32.71 (10.57)	16.42 (3.19)	11.40 (3.66)
Yes	360	35.12 (9.31)	35.12 (9.31)	35.12 (9.31)	17.10 (2.86)	10.53 (3.24)
*t*-value		−3.35***	−2.53**	1.99*	−3.11**	3.47***
**Co-residence**						
Living alone	56	31.52 (10.69)	11.91 (2.69)	9.02 (2.81)	16.52 (3.06)	11.89 (3.82)
With spouse only	304	35.78 (8.90)	12.57 (2.30)	7.71 (2.55)	17.21 (2.82)	10.28 (3.07)
With children only	98	31.61 (11.20)	11.82 (2.91)	8.70 (2.88)	16.15 (3.26)	11.65 (3.64)
With spouse and children	292	33.01 (10.28)	12.06 (2.68)	8.18 (2.90)	16.46 (3.16)	11.33 (3.67)
With others	15	33.93 (12.10)	11.93 (2.79)	8.27 (3.77)	17.33 (3.20)	11.07 (3.65)
*F*-value		5.39***	2.51*	4.25**	3.55**	5.80***

*p < 0.05; **p < 0.01; ***p < 0.001.

### Living Arrangements and SWB

Empty-nest elders had significantly higher SWB than did non-empty-nest elders [*t*(763) = 3.353, *p* < 0.001]. Participants living with a spouse only had the highest SWB (*M* = 35.78, *SD* = 8.90), which was significantly higher than that among other elders. Participants living alone had the lowest SWB (*M* = 31.52, *SD* = 10.69) among all participants, however, it did not significantly differ from the SWB of participants living with children only (Table 3). Thus, having a spouse may have a larger effect on elders’ SWB than having children.

### Interaction Effect of Living Regions and Living Arrangements on SWB

In the interaction model of living regions and “co-residence,” the main effects of the two variables remained significant and no interaction effect was found. In the interaction model of living regions and “empty-nest,” the main effect of living regions remained significant, while the main effect of empty-nest was non-significant. No interaction effect was found (Table 4). These results suggest that the prior findings – empty-nest elders having significantly higher SWB than non-empty-nest elders – was affected by the disproportion rate of empty-nest elders/non-empty-nest elders among living regions since there were more empty-nest elders in urban areas than in rural areas, and urban elders had higher SWB than did rural elders.

**TABLE 4:** Interaction effect of living regions and living arrangements on subjective well-being.

Dependent variable: subjective well being	Source		Type III sum of square	df	Mean square	F	Sig.
	Model 1		8283.72	14	591.69	6.42	0.000
		Intercept	252367.40	1	252367.40	2738.23	0.000
		Living regions	1975.15	2	987.57	10.72	0.000
		Co-residence	1224.12	4	306.03	3.32	0.010
		Living regions * Co-residence	452.50	8	56.56	0.61	0.767
		Error	69123.46	750	92.17		
		Total	953606.00	765			
		Corrected total	77407.18	764			
	
	Model 2		7098.31	5	1419.66	15.33	0.000
		Intercept	800748.82	1	800748.82	8644.26	0.000
		Living regions	5672.62	2	2836.31	30.62	0.000
		Empty-nest	230.04	1	230.04	2.48	0.115
		Living regions * Empty-nest	31.13	2	15.57	0.17	0.845
		Error	70308.87	759	92.63		
		Total	953606.00	765			
		Corrected total	77407.18	764			

### Predicting SWB by Regression Models

As shown in Table 5, in Model 0 younger elders, those with a junior high school or high school education, and those with fewer illnesses tended to have higher SWB than did their counterparts.

**TABLE 5:** Unstandardized ordinary least squares regression estimates subjective well-being (*N* = 765).

	Reference variable	Model 0	Model 1	Model 2	Model 3	Model 4	Model 5	Model 6	Model 7
**Constant**		16.87***	16.00***	10.96*	7.89	10.51**	10.22*	6.21	9.01*
**Sex**	Women	0.18	0.23	0.09	0.78	1.10	0.06	0.73	1.04
**Age**		0.25***	0.25***	0.30***	0.18***	0.14**	0.30***	0.19***	0.14*
**Religion**	No	1.55	1.56	1.36	1.71*	1.62	1.50	1.78*	1.71*
**Education**	Elementary and below								
Junior high		2.36**	2.13*	1.84*	1.81*	1.38	1.83*	1.82*	1.39
High		2.47*	2.19	1.85	1.00	0.67	1.74	0.92	0.61
College and above		1.55	1.29	0.95	0.09	−0.49	0.82	0.01	−0.56
**Previous vocation**	No vocation								
Farmer		−1.44	−1.17	−1.25	−0.53	−0.28	−1.17	−0.51	−0.23
Worker		3.03	3.06	3.04	0.88	0.09	3.23	1.10	0.33
Others		3.89	4.00	3.69	0.85	0.45	3.84	1.01	0.64
Number of children		−0.05	−0.03	−0.07	0.10	0.47	−0.05	0.10	0.48
**Health status**									
Number of illness		−0.93*	−0.95*	−0.98*	−0.51	−0.70*	−0.97*	−0.50	−0.68
Chronical illness	No	−1.80	−1.81	−1.61	−1.36	−1.13	−1.58	−1.34	−1.11
Critical illness	No	−1.25	−1.24	−1.30	−1.21	−1.16	−1.30	−1.21	−1.17
Marital status	Unmarried			2.75**	1.59	2.02**			
**Financial status**									
Income per month					0.49	0.02		0.46	0.00
Retirement pension	No				−0.37	−0.38		−0.40	−0.43
Income adequacy					4.93***	4.99***		4.92***	4.98***
**Empty-nest**	Non−empty−nest		1.67*	1.40*	0.51	0.48			
**Co-residence**	Living alone								
With spouse only							5.07***	3.81**	3.97**
With children only							1.04	1.59	1.30
With spouse and children							2.98*	2.71*	2.95*
With others							3.43	3.59	3.68
**Living place**	Rural areas								
Small counties						0.83			0.87
Large cities						4.47***			4.44***
**Adj. R square**		0.138	0.144	0.153	0.30	0.31	0.158	0.300	0.31

*p < 0.05; **p < 0.01; ***p < 0.001.

In Model 1, empty-nest was introduced, and the results were consistent with the result that empty-nest elders had higher SWB than did non-empty-nest elders. After current marital status was controlled in Model 2, empty-nest elders still had higher SWB than did non-empty-nest elders, however, the significance was marginal (*p* = 0.048). After financial status was further controlled in Model 3, being an empty-nester could no longer significantly predict SWB (*p* = 0.434), however, financial status, especially perceived income adequacy, positively predicted SWB (*p* < 0.001). Finally, living region was entered, revealing that elders living in large cities had better SWB than did other elders.

Model 5 revealed that elders living with a spouse (including living with a spouse only and living with a spouse and children) had better SWB than did other elders. After financial status was further controlled in Model 6, living with a spouse still significantly predicted SWB. Finally, living regions was introduced in Model 7, also revealing that elders living in large cities had better SWB than did other elders.

## Discussion

### Main Findings

First, participants were happy on average. This result was consistent with Diener and Diener (1996) finding that most people are happy. The more important question here may be *who* is happier. In this study, before sociodemographic variables were controlled, empty-nest elders had higher SWB than did non-empty-nest elders. Some possible reasons may be that more empty-nest elders were living in big cities and were currently married than were non-empty-nest elders, empty-nest elders had better financial status than did non-empty-nest elders, etc. However, after controlling for living region, marital status, financial status, and other variables, being an empty-nester could not significantly predict SWB anymore. Further, the results did not show that empty-nest elders were unhappier than other elders, which indicated that being an empty-nester may not be as negative as portrayed in the mass media, even though the empty-nest living arrangement runs in the opposite direction of traditional Chinese culture; i.e., elders living with children (Sung, 2001; Zeng and George, 2010).

According to post-modernism, there are different kinds of families: single-parent families, adoptive families, gay and lesbian families, and so on (Elkind, 1995), including empty-nest families. However, family life cycle theory does not allow for alternative family lifestyles, instead, it assumes there is a perfect, standard, and normal family style (Derrick and Lehfeld, 1980). On the contrary, criticists (Derrick and Lehfeld, 1980) and many studies (e.g., Bouchard, 2014; Zhang, 2019), including this study, showed that other types of families, such as empty-nest families and empty-nest elders could also be happy. As Ye and Huang (2015) mentioned, an empty-nest is not that “empty.”

Elkind (1995) noted that the value of the postmodern permeable family is that of autonomy, the importance of individual choice, and one’s personal life journey. These are also the values of an individualized society (Yan, 2009). Although traditional Chinese society values filial piety, regarding a big family as ideal and considering four generations living together as the utmost happiness (Hsu, 1948), Chinese society is individualizing and Chinese families are diversifying (Yan, 2009). The living arrangements of Chinese elders are partially becoming Westernized, and most are adapting well (Wang et al., 2014). In other words, an increasing number of elders are unwilling to live with children and can live independently and happily (Miller, 2007).

Another component of social individualization is that the marital relationship replaced the parent–child relationship as the most important relationship in families (Yan, 2009). This study found that elders living with a spouse (regardless of living with children) had higher SWB than did other elders, including before and after other variables were controlled for. This result was consistent with Weissman and Russell (2016) finding that in an individualistic country – the United States – older women living with a spouse tended to have better health than did women living alone or with children. This result also indicates that the concept of empty-nest elders was unreasonable since it did not separate married and unmarried elders. It focused on the role of children but neglected the role of social change and the growing importance of having a spouse. However, with social individualization, it may be an inevitable trend for elders to live apart from children and rely more on a spouse. Consequently, researchers should focus more on how to develop non-family care policies and programs that assist with improving the SWB of elders living without a spouse.

Moreover, although being an empty-nester could not predict elders’ SWB, living region could. Elders living in large cities had the highest SWB compared with elders living in small counties or rural areas. One possible reason is that there is large income gap between urban and rural Chinese (Du, 2013; Xing, 2014). Wu (2013) found an increasing gap in pension levels between urban and rural areas. In this study, many urban elders had a retirement pension, while only 8% of rural elders had one. Statistics from the National Bureau of Statistics (2010) showed that the ratio of urban per capita annual income to that of rural households increased from 2.2:1 in 1990 to 3.3:1 in 2009.

Further, perceived income adequacy significantly predicted SWB. In other words, it is not how much money an elder had that matters for his or her SWB, but whether the elder felt the income was adequate. The results concerning perceived income were inconsistent with those of Western researchers, such as Stoller and Stoller (2003) finding that elderly people generally find their incomes to be adequate, even when those incomes are relatively low. This may suggest that, compared to elders in developed countries, Chinese rural elders’ income is too low to meet their needs.

In addition, after controlling for financial status, living region still significantly predicted elders’ SWB. This implies that, besides the objective and subjective income gap, other gaps between rural and urban areas may also affected elders’ SWB. Zhang and Goza (2006) stated that elders in urban areas have many possibilities, while elders in rural areas lack options. For example, although all the participants in this study had medical insurance, the system itself differed: the Basic Health Insurance for Urban Residents differs substantially from the New Rural Cooperative Medical Care System (Liu and Darimont, 2013). The healthcare conditions of rural residents are typically much worse than those of their urban cohorts (Zhang and Kanbur, 2005). Rural residents, especially poor rural residents and most farmers, are also still troubled by “poverty caused by illness” (Zhang and Wen, 2016).

Finally, although the results revealed that empty-nest elders were not unhappy, there may be some mechanisms by which living without children can affect elders’ SWB, such as inter-generational support (Guo et al., 2009; Zhou et al., 2015), their role as grandparents (Sun, 2012), coping style (Xie et al., 2010), and religiosity and perceived control (Jackson and Bergeman, 2011).

### Implications

Contrary to the media’s negative views on empty-nest elders, this study found that empty-nest elders were not unhappier than non-empty-nest elders. Instead, elders living without a spouse and rural elders had a high risk of being unhappy. This suggests that researchers and policymakers should shift their attentions from empty-nest families to the widowed and rural elders, especially rural widowed elders. For example, the government could try to improve non-family care policies and programs that assist elders living without a spouse, try to reduce the urban–rural gap in income by increasing the ratio and amount of retirement pension for rural residents, and try to unify the urban and rural medical insurance policies.

### Limitations

This study examined the effect of living regions on Chinese elders’ SWB, however, it was impossible for us to enroll participants from all the provinces in China. Future researchers could create a “happiness map” of China to clarify the regional differences in SWB. In fact, Diener and his colleagues suggested creating a national happiness index (Diener and Seligman, 2006; Diener et al., 2008). Second, future researchers could apply qualitative methods to better understand the reasons behind living arrangement and its impact on SWB; for example, why elders choose to live independently, why some married elders live alone without a spouse, and how empty-nest elders maintain their happiness. Third, this study only controlled for some socioeconomic variables that are highly related to SWB (Diener, 1999), including older adults’ SWB (Pinquart and Sorensen, 2000). It was impossible and unnecessary for us to control all related variables, however, future research should consider other control variables that may also affect elders’ SWB, for example, housing (Tran and Van Vu, 2018) and economic inequality (Tran et al., 2018).

### Strengths

Despite these limitations, this study has several meaningful contributions. First, it adds to the literature on underprivileged Chinese families, which are conducted far less compared to studies about Western families (Shek, 2006). More importantly, this study provided support that empty-nesters are not “empty,” and the negative portrayal by the media and some prior researchers may be incorrect. This study illuminates a new perspective to understand empty-nest elders in China. This 1990s label is impropriate, and married and unmarried empty-nest elders should not be simply grouped together. Finally, this study informs researchers and policymakers to focus more on the influences of society on elders instead of family, such as the unbalanced development between rural and urban areas.

## Conclusion

With aging, the one-child policy, and migration, being an empty-nester may be an inevitable trend for Chinese elders. Contrary to the negative descriptions of empty-nest elders, the study found that empty-nest elders were not unhappier than non-empty-nest elders. The impact of children on Chinese elders’ SWB has thus been overestimated. Although traditional China values filial piety, modern Chinese society is individualizing and spouses are replacing children as the most important familial relationships, i.e., elders living with a spouse had higher SWB than did other elders. Thus, instead of encouraging children to care for their aging parents, the central government should focus more on how to develop non-family care policies and programs that assist elders living without a spouse.

Moreover, unbalanced development of different regions had a larger effect on elders’ SWB than did living arrangement. Living region could significantly predict elders’ SWB, regardless of elders’ financial status and other variables. This indicates that urban–rural gaps in income, perceived income, and other aspects may have a strong impact on elders’ SWB. However, it is difficult for elders to choose where they live, therefore it is up to the government to increase the SWB of rural elders by reducing regional differences and addressing the urban–rural gap.

## Data Availability Statement

The datasets generated for this study are available on request to the corresponding author.

## Ethics Statement

Ethical review and approval was not required for the study on human participants in accordance with the local legislation and institutional requirements. Written informed consent for participation was not required for this study in accordance with the national legislation and the institutional requirements.

## Author Contributions

YZ contributed on the design of the study, data process and data analysis, and writing of the manuscript.

## Conflict of Interest

The author declares that the research was conducted in the absence of any commercial or financial relationships that could be construed as a potential conflict of interest.

## References

[B1] Andrews F. M. Robinson J. P. (1991). “Measures of subjective well-being,” in *Measures of Personality and Social Psychological Attitudes*, eds Robinson J. P. Shaver P. R. Wrightsman L. S. (San Diego, CA: Academic Press, Inc), 61–114. 10.1016/b978-0-12-590241-0.50007-1

[B2] Ao X. Jiang D. Zhao Z. (2015). The impact of rural-urban migration on the health of the left-behind parents. *China Economic Rev.* 37 126–139. 10.1016/j.chieco.2015.09.007

[B3] Beck U. Beck-Gernsheim E. (2001). *Individualizaion: Institutionalized Individualism and its Social and Political Consequences.* London: SAGE.

[B4] Borland D. C. (1982). A cohort analysis approach to the empty-nest syndrome among three ethnic groups of women: a theoretical position. *J. Marriage Fam.* 44 117–129.

[B5] Bouchard G. (2014). How do parents react when their children leave home? An integrative review. *J. Adult Dev.* 21 69–79. 10.1007/s10804-013-9180-8

[B6] Cheng P. Jin Y. Sun H. Tang Z. Zhang C. Chen Y., et al. (2015). Disparities in prevalence and risk indicators of loneliness between rural empty nest and non-empty nest older adults in Chizhou, China. *Geriatr. Gerontol. Int.* 15 356–364. 10.1111/ggi.12277 24629147

[B7] CHINADAILY (2015). *Floods Reveal Plight of “Left Behind” Seniors.* Available online at: http://www.chinadaily.com.cn/china/2015-06/17/content_21034087.htm (accessed September 8, 2016).

[B8] Deng J. L. Hu J. M. Wu W. L. Dong B. R. Wu H. M. (2010). Subjective well-being, social support, and age-related functioning among the very old in China. *Int. J. Geriatr. Psychiatry* 25 697–703. 10.1002/gps.2410 20033902

[B9] Derrick F. W. Lehfeld A. K. (1980). The family life cycle: an alternative approach. *J. Consum. Res.* 7 214–217. 10.1086/208809

[B10] Diener E. (1999). Subjective well-being: three decades of progress. *Psychol. Bull.* 125 276–302. 10.1037/0033-2909.125.2.276 7630576

[B11] Diener E. Diener C. (1996). Most people are happy. *Psychol. Sci.* 7 181–185. 10.1111/j.1467-9280.1996.tb00354.x

[B12] Diener E. Kesebir P. Lucas R. (2008). Benefits of accounts of well-being - for societies and for psychological science. *Appl. Psychol.* 57(Suppl. 1), 37–53. 10.1111/j.1464-0597.2008.00353.x 27226808

[B13] Diener E. Seligman M. E. P. (2006). Measure for measure: the case for a national well-being index. *Sci. Spirit* 17 36–37. 10.3200/sspt.17.2.36-37

[B14] Du P. (2013). Intergenerational solidarity and old-age support for the social inclusion of elders in Mainland China: the changing roles of family and government. *Ageing Soc.* 33 44–63. 10.1017/S0144686x12000773

[B15] Duvall E. M. (1957). *Family Development.* New York, NY: J. B. Lippincott Co.

[B16] eChinacities (2014). *Those Left Behind: China’s Deserted Villages.* Available online at: http://www.echinacities.com/china-media/Those-Left-Behind-Chinas-Deserted-Villages (accessed September 8, 2016)

[B17] Elkind D. (1995). The family in the postmodern age. *Natl. Forum* 75 24–28. 10.4324/9781315132006-7

[B18] Fang E. F. Scheibye-Knudsen M. Jahn H. J. Li J. Ling L. Guo H. (2015). A research agenda for aging in China in the 21st century. *Ageing Res. Rev.* 24 197–205. 10.1016/j.arr.2015.08.003 26304837 PMC5179143

[B19] Guo M. Aranda M. P. Silverstein M. (2009). The impact of out-migration on the inter-generational support and psychological wellbeing of older adults in rural China. *Ageing Soc.* 29 1085–1104. 10.1017/S0144686X0900871X

[B20] Hsu F. L. K. (1948). *Under the Ancestors’ Shadow: Chinese Culture and Personality.* New York, NY: Columbia University Press.

[B21] Jackson B. R. Bergeman C. S. (2011). How does religiosity enhance well-being? The role of perceived control. *Psychol. Relig. Spiritual.* 3 149–161. 10.1037/a0021597 PMC4029596 24860640

[B22] Kozma A. Stones M. J. (1983). Predictors of happiness. *J. Gerontol.* 38 626–628. 6886321 10.1093/geronj/38.5.626

[B23] Li C. Chi I. Zhang X. Cheng Z. Zhang L. Chen G. (2014). Urban and rural factors associated with life satisfaction among older Chinese adults. *Aging Ment. Health* 19 947–954. 10.1080/13607863.2014.977767 25407598

[B24] Lin M. Liu Y. Zhan G. (2009). Empirical study on the urban elderly people of “Empty Nest” and their loneliness (in Chinese). *Mod. Prev. Med.* 36 77–80.

[B25] Liu D. Darimont B. (2013). The health care system of the People’s Republic of China: between privatization and public health care. *Int. Soc. Secur. Rev.* 66 97–116. 10.1111/issr.12004

[B26] Liu L. J. Sun X. Zhang C. L. Guo Q. (2007). Health-care utilization among empty-nesters in the rural area of a mountainous county in China. *Public Health Rep.* 122 407–413. 10.1177/003335490712200315 17518313 PMC1847485

[B27] Liu L. L. Guo Y. Dou J. (2014). Comparision of elder’s psychology in three common residential model (in Chinese). *Chin. J. Gerontol.* 34 1917–1919.

[B28] Ma Y. Fu H. Wang J. Fan L. Zheng J. Chen R. (2012). Study on the prevalence and risk factors of depressive symptoms among “empty-nest” and “non-empty-nest” elderly in four provinces and cities in China (in Chinese). *Zhonghua Liu Xing Bing Xue Za Zhi* 33 478–482. 22883173

[B29] Miller E. T. (2007). “Living independently is good”: residence patterns in rural north China reconsidered. *Care Manage. J.* 8 26–32. 10.1891/152109807780494113 17491448

[B30] Mitchell B. A. Lovegreen L. D. (2009). The empty nest syndrome in midlife families: a multimethod exploration of parental gender differences and cultural dynamics. *J. Fam. Issues* 30 1651–1670. 10.1177/0192513x09339020

[B31] National Bureau of Statistics (2010). Available online at: http://data.stats.gov.cn/english/easyquery.htm?cn (C01 (accessed May 6, 2016).

[B32] Ng A. C. Y. Phillips D. R. Lee W. K. (2002). Persistence and challenges to filial piety and informal support of older persons in a modern Chinese society. *J. Aging Studies* 16 135–153. 10.1016/S0890-4065(02)00040-3

[B33] Pinquart M. Sorensen S. (2000). Influences of socioeconomic status, social network, and competence on subjective well-being in later life: a meta-analysis. *Psychol. Aging* 15 187–224. 10.1037/0882-7974.15.2.187 10879576

[B34] Poulin J. Houser L. Deng R. (2014). The life satisfaction of elderly Chinese widows living alone and those living with adult children. *China J. Soc. Work* 7 119–130. 10.1080/17525098.2014.921210

[B35] Ren Q. Treiman D. J. (2015). Living arrangements of the elderly in china and consequences for their emotional well-being. *Chin. Sociol. Rev.* 47 255–286. 10.1080/21620555.2015.1032162

[B36] Shek D. T. L. (2006). Chinese family research: puzzles, progress, paradigms, and policy implications. *J. Fam. Issues* 27 275–284. 10.1177/0192513x05283508

[B37] Silverstein M. Cong Z. Li S. (2006). Intergenerational transfers and living arrangements of older people in rural China: consequences for psychological well-being. *J. Gerontol. B Psychol. Sci. Soc. Sci.* 61 S256–S266. 10.1093/geronb/61.5.S256 16960239

[B38] Stoller M. A. Stoller E. P. (2003). Perceived income adequacy among elderly retirees. *J. Appl. Gerontol.* 22 230–251. 10.1177/0733464803022002004 644418

[B39] Sun J. (2012). Chinese older adults taking care of grandchildren: practices and policies for productive aging. *Ageing Int.* 38 58–70. 10.1007/s12126-012-9161-4

[B40] Sun J. J. (2010). Migration of adult children and its impact on family intergenerational relationship of the rural China (in Chinese). *Popul. J.* 1 28–33.

[B41] Sung K. (2001). Family support for the elderly in Korea. *J. Aging Soc. Policy* 12 65–79. 10.1300/J031v12n04 11799915

[B42] The State Council of China (2017). *China Issues Five-Year Plan on Elderly Care.* Available online at: http://www.gov.cn/zhengce/content/2017-03/06/content_5173930.htm (accessed March 23, 2020).

[B43] Tian T. Chen Y. Zhu J. Liu P. (2015). Effect of air pollution and rural-urban difference on mental health of the elderly in china. *Iran J. Public Health* 44 1084–1094. 26587472 PMC4645728

[B44] Tran T. Q. Nguyen C. V. Van Vu H. (2018). Does economic inequality affect the quality of life of older people in rural vietnam? *J. Happiness Stud.* 19 781–799. 10.1007/s10902-017-9851-4 20831822

[B45] Tran T. Q. Van Vu H. (2018). A microeconometric analysis of housing and life satisfaction among the Vietnamese elderly. *Qual. Quant.* 52 849–867. 10.1007/s11135-017-0492-9

[B46] United Nations (2013). *International Migration Report 2013.* New York, NY: United Nations.

[B47] Wang J. Chen T. Han B. (2014). Does co-residence with adult children associate with better psychological well-being among the oldest old in China? *Aging Ment. Health* 18 232–239. 10.1080/13607863.2013.837143 24053437 PMC4158609

[B48] Wang X. (1995). A brief analysis of the basic characteristics of urban elders in “empty nest family” (in Chinese). *Urban Probl.* 19–24.

[B49] Wang X. Shang X. Xu L. (2011). Subjective well-being poverty of the elderly population in China. *Soc. Policy Adm.* 45 714–731. 10.1111/j.1467-9515.2011.00804.x

[B50] Weissman J. D. Russell D. (2016). Relationships between living arrangements and health status among older adults in the united states, 2009-2014: findings from the national health interview survey. *J. Appl. Gerontol.* 37 7–25. 10.1177/0733464816655439 27353832

[B51] Wu L. (2013). Inequality of pension arrangements among different segments of the labor force in china. *J. Aging Soc. Policy* 25 181–196. 10.1080/08959420.2012.735159 23570510

[B52] Xie L. Q. Zhang J. P. Peng F. Jiao N. N. (2010). Prevalence and related influencing factors of depressive symptoms for empty-nest elderly living in the rural area of YongZhou, China. *Arch. Gerontol. Geriatr.* 50 24–29. 10.1016/j.archger.2009.01.003 19217674

[B53] Xing C. (2014). Migration, self-selection and income distributions. *Econ. Transit.* 22 539–576. 10.1111/ecot.12041

[B54] XINHUA (2017). *Full Text of Xi Jinping’s Report at 19th CPC National Congress.* Available online at: http://www.xinhuanet.com/english/special/2017-11/03/c_136725942.htm (accessed May 9, 2018).

[B55] Xu Q. Li J. Yu X. (2014). Continuity and change in chinese marriage and the family. *Chin. Sociol. Rev.* 47 30–56. 10.2753/CSA2162-0555470102.2014.11082909

[B56] Yan Y. (2009). *The Individualization of Chinese Society.* Oxford: Berg Publishers.

[B57] Yan Y. (2011). The individualization of the family in rural china. *Boundary 2* 38 203–229. 10.1215/01903659-1262590 28343403

[B58] Ye M. Huang W. (2015). Empty nests are not that empty: reestimating the effect of empty-nest on older adults’ subjective quality of life in mainland china. *Gerontologist* 55(Suppl. 2):445 10.1093/geront/gnv191.07

[B59] Zeng Y. George L. K. (2010). “Population ageing and old-age insurance in china,” in *The SAGE Handbook of Social Gerontology*, eds Dannefer D. Phillipson C. (Los Angeles, CA: SAGE), 421.

[B60] Zhai Y. Yi H. Shen W. Xiao Y. Fan H. He F. (2015). association of empty nest with depressive symptom in a chinese elderly population: a cross-sectional study. *J. Affect. Disord.* 187 218–223. 10.1016/j.jad.2015.08.031 26342917

[B61] Zhang C. (2019). Are children from divorced single-parent families disadvantaged? New evidence from the china family panel studies. *Chin. Sociol. Rev.* 52 84–114. 10.1080/21620555.2019.1654366

[B62] Zhang D. Wen X. (2016). Strategy research on improving rural medical and health services under the background of new-type urbanization (in Chinese). *Dev. Res.* 68–74.

[B63] Zhang X. Kanbur R. (2005). Spatial inequality in education and health care in China. *China Econ. Rev.* 16 189–204. 10.1016/j.chieco.2005.02.002

[B64] Zhang Y. Goza F. W. (2006). Who will care for the elderly in China? *J. Aging Stud.* 20 151–164. 10.1016/j.jaging.2005.07.002

[B65] Zhou B. Chen K. Wang J. Wang H. Zhang S. Zheng W. (2010). Quality of life and related factors in the older rural and urban Chinese populations in Zhejiang province. *J. Appl. Gerontol.* 30 199–225. 10.1177/0733464810361346

[B66] Zhou Y. Zhou L. Fu C. Wang Y. Liu Q. Wu H. (2015). Socio-economic factors related with the subjective well-being of the rural elderly people living independently in China. *Int. J. Equity in Health* 14 1–9. 10.1186/s12939-015-0136-4 25595196 PMC4299131

